# Revisiting the Venoarteriolar Reflex–Further Insights from Upper Limb Dependency in Healthy Subjects

**DOI:** 10.3390/biology13090715

**Published:** 2024-09-12

**Authors:** Henrique Silva, Carlota Rezendes

**Affiliations:** 1 Research Institute for Medicines (iMed.ULisboa), Faculdade de Farmácia, Universidade de Lisboa, Av. Prof. Gama Pinto, 1649-003 Lisbon, Portugal; 2 Department of Pharmacy, Pharmacology and Health Technologies, Faculdade de Farmácia, Universidade de Lisboa, Av. Prof. Gama Pinto, 1649-003 Lisbon, Portugal; carlotarezendes@edu.ulisboa.pt; 3 Biophysics and Biomedical Engineering Institute (IBEB), Faculdade de Ciências, Universidade de Lisboa, Campo Grande, 1749-016 Lisbon, Portugal

**Keywords:** venoarteriolar reflex, upper limb, contralateral response, skin perfusion, sympathetic nervous system, photoplethysmography, electrodermal activity

## Abstract

**Simple Summary:**

The venoarteriolar reflex (VAR) consists of a local vasoconstriction occurring in response to an increase in venous transmural pressure. Its underlying mechanisms, including its impact on contralateral perfusion, still need clarification. In this study we assessed VAR in a group of healthy subjects by performing a unilateral limb dependency procedure. During dependency skin perfusion decreased significantly in both the dependent and the contralateral hands. Cutaneous sympathetic activity increased during dependency and upon returning to the initial position. These results corroborate previous reports that unilateral limb dependency impacts contralateral perfusion and suggest the participation of the sympathetic nervous system. This sympathetic activation seems to occur in response to the postural change itself and does not seem to be related to the VAR.

**Abstract:**

The venoarteriolar reflex (VAR) is described as a vasoconstriction occurring in response to an increase in venous transmural pressure. Its underlying mechanisms are still not clarified, particularly the neural pathway that supposedly evokes this reflex. In addition, recent studies have shown that the postural maneuvers that evoke VAR also produce a decrease in contralateral perfusion, which is also poorly understood. Our study aimed to explore the contralateral response to unilateral upper limb dependency and its underlying mechanisms. Fifteen young, healthy subjects (24.1 ± 5.8 y.o.) participated in this study after giving informed consent. While seated, subjects remained for 7 min with both arms at heart level (baseline), after which a random hand was placed 40 cm below the heart level for 5 min (dependency) before resuming the initial position for another 7 min (recovery). Skin perfusion was assessed bilaterally with photoplethysmography, and electrodermal activity (EDA) was assessed in the contralateral hand. During hand dependency, perfusion decreased significantly in both limbs, although it was more pronounced in the dependent limb, corroborating previous reports that unilateral limb dependency evokes a decrease in contralateral perfusion. Transient EDA peaks were detected in the first seconds of the dependency and recovery phases. These results support the participation of the sympathetic nervous system as a mechanism regulating contralateral perfusion during unilateral limb dependency. This sympathetic activation is probably attributed to the postural changes themselves and is likely not related to the VAR.

## 1. Introduction

The venoarteriolar reflex (VAR), also known as postural vasoconstriction, is an important homeostatic response that regulates the perfusion pressure of dependent regions of the body, particularly the limbs [1]. It is currently defined as an arteriolar vasoconstriction that occurs in response to an increase in venous pressure (above 25 mmHg in the limbs). The resulting decrease in perfusion prevents the increase in the hydrostatic capillary pressure and, consequently, the increase in the net filtration pressure and the formation of edema. Although more relevant in the limbs, the VAR has also been described in the head and neck regions.

Current experimental evidence suggests that the VAR is a neural reflex, even though the neural pathway involved is still poorly understood. The observations that the VAR is abolished by local anesthetics (lidocaine, prilocaine or both) [2,3,4,5,6] suggest that this response is consistent with a local axon reflex, a type of reflex that is also involved in regulating the activity of sweat glands. In this hypothesis, venous wall distension generates nerve impulses that are propagated orthodromically along axon arborizations until a branching point is reached, from which the impulses are then propagated antidromically to arterioles [7]. The hypothesis of whether nerve impulses from veins also reach the central nervous system is also under discussion. The majority of published studies have shown that the VAR is maintained in areas distal to nerve lesions in paraplegic [8,9] and tetraplegic patients [10], suggesting that this reflex occurs without integration of afferent nerve signals by the brainstem, although spinal integration may occur. The dual ‘sensory–effector’ role of nerve fibers allowing the communication between veins and neighboring arterioles was initially attributed to post-ganglionic sympathetic fibers [11,12,13]. However, recent studies have demonstrated that the sympathetic adrenergic nerve fibers are not involved in the VAR [3,14].

Besides this apparent neural component, the VAR may be occurring simultaneously with other perfusion-regulating mechanisms, namely, with the myogenic activity. This notion stems from the comparison between the different experimental approaches that have been used to evoke this reflex. The VAR is usually evoked by inducing venous congestion (i.e., ‘loading’) through proximal venous occlusion [2,15,16,17] or by changing the position of a limb [18,19]. Changes in the whole-body posture (e.g., changing from a supine or seated position to standing; body tilting) [20] and the application of whole/lower-body negative pressure [21] are also known to evoke the VAR. However, since they also induce the baroreceptor reflex, they cannot assess the VAR individually. Proximal venous occlusion is performed with the limb in a static position by inflating a cuff around the proximal region of a limb to 25 mmHg or higher. This value is well below the diastolic blood pressure, which induces venous occlusion while guaranteeing arterial patency. The resulting venous congestion then initiates the VAR [2]. In contrast, postural modifications typically consist of placing a given limb in a dependent position (i.e., below the heart level) so that the increase in the hydrostatic effect induces venous pooling in distal territories. However, limb dependency also causes arterial congestion, which causes a direct myogenic vasoconstriction [2,3]. In a minority of studies, the experimental approach was to suppress the VAR by venous ‘unloading’, that is to change a limb from a dependent to a nondependent position [1,22,23], which also induces myogenic vasodilation. As such, the VAR may occur solely or simultaneously with a myogenic response, depending on the specific nature of the experimental maneuver.

The existence of a systemic component to the VAR has also been the subject of some speculation, with recent studies suggesting that this reflex might be part of a broader homeostatic cardiovascular response [19,24]. In fact, when the VAR was evoked by unilateral foot dependency, a significant decrease in perfusion in the contralateral foot was also observed, which was attributed to a neurogenic-mediated vasoconstriction. With regards to the upper limbs, this contralateral response has not been well characterized. Considering that the lower limbs are subjected to a higher hydrostatic effect than the upper limbs [25], it is logical that the magnitude of the VAR and of the contralateral response would be lower in the latter region.

Our study aimed to explore the magnitude of the VAR and contralateral response to unilateral upper limb dependency in healthy subjects. We hypothesized that the presence of a contralateral vascular response would be mediated by the sympathetic nervous system. We also provide a thorough discussion into the probable physiological mechanisms that underlie both phenomena based on a critical analysis of the most relevant studies published thus far.

## 2. Materials and Methods

### 2.1. Participants

Fifteen young healthy subjects (24.1 ± 5.8 years old, 9 females and 6 males) were included in this study after giving informed written consent. The defined inclusion criteria were male or female (non-pregnant), between 18 and 35 years old, non-obese (body mass index < 30 kg/m^2^), and normotensive (blood pressure < 130/90 mmHg). The defined exclusion criteria were a current or past history of cardiovascular, metabolic, neurologic or psychiatric diseases, and taking vasoactive medications (except contraceptives) or dietary supplements. Subjects were asked to refrain from drinking caffeinated beverages and from performing physical exercise 12 h prior to the procedures. Also, subjects were instructed to fast for 2–4 h before performing the procedures to minimize any potential effects on sympathetic drive and endothelial activity [26,27,28]. Table 1 summarizes the characteristics of these subjects. The study was approved by the local ethics committee and followed the recommendations of the Declaration of Helsinki and subsequent amendments for studies conducted in human subjects [29].

### 2.2. Procedures

**Figure 1:**
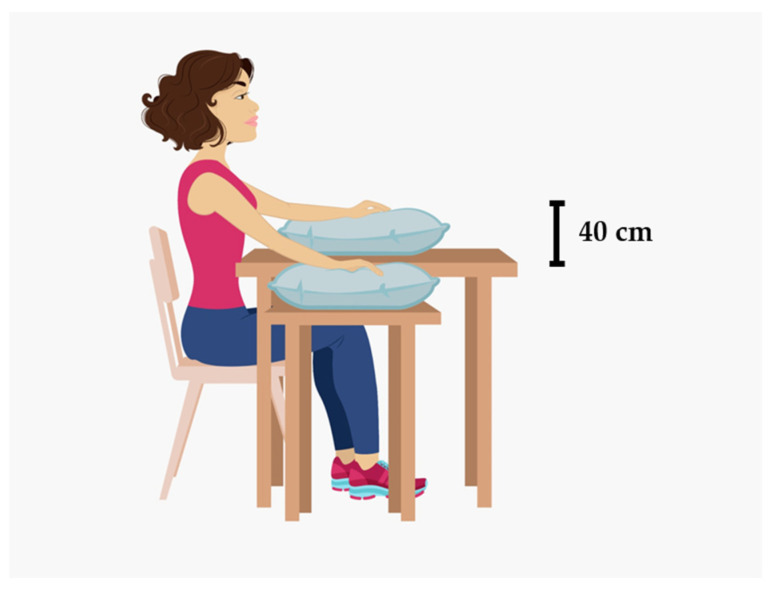
Illustration of the posture adopted by the subjects during the dependency phase of the experimental procedure.

Procedures were carried out in a room with controlled temperature and humidity (25 ± 1 °C, 40–60%). Subjects were lightly clothed and acclimatized to the room conditions prior to the procedure for 20 min. Blood pressure was measured in a randomly chosen arm at the heart level and in the dependent position (40 cm below the heart level) to ensure that subjects were normotensive. During the procedure, subjects were sitting upright with both feet on the floor and with both arms in extension and in the supine position at the heart level and supported by a table in front of them. The procedure consisted of three phases as follows: Subjects remained with both arms at the heart level for 7 min (phase I—baseline); then, the subjects actively placed one random arm approximately 40 cm below heart level for 5 min, again with physical support, while the contralateral arm remained unmoved (phase II—dependency). Finally, the initial position was resumed for a further 7 min (phase III—recovery). Figure 1 illustrates the posture adopted by the subjects during the dependency phase of the procedure.

### 2.3. Technologies

Several physiological variables were acquired during this procedure. Skin blood flow was recorded with reflection photoplethysmography (PPG) sensors taped to the palmar surface of the distal phalanx of the second finger of both hands. The sensors emitted a green light with a 530 nm wavelength and had a 2.3 mm separation between the LED and receptor. PPG is a low-cost optical technique that quantifies microcirculatory perfusion. It quantifies the amount of visible light reflected by the microvasculature, which is proportional to the circulating blood volume [30]. The PPG waveform is composed of two components: a direct current (DC) and an alternating current (AC). The DC component corresponds to the amount of signal reflected by the illuminated tissues, which depends on the amount of blood flow contained in blood vessels, as well as on the characteristics of the tissue itself. In contrast, the AC component is related to the changes in blood volume created by arterial pulses that follow each cardiac cycle. Recently, it has been proposed that changes in the capillary diameter caused by fluctuations in arterial transmural pressure [31] and changes in the red blood cell orientation in these capillaries [32] are also responsible for certain features of the PPG waveform.

Skin temperature was recorded to assess whether changes in skin blood flow were related to thermoregulation, as suggested by guidelines of cutaneous perfusion quantification [33]. Also, it has been demonstrated that the magnitude of the VAR depends on skin temperature [34]. Negative temperature coefficient (NTC) thermistors were placed on the palmar surface of the middle phalanx of the second fingers of both hands, inferior to the PPG sensors. In NTC thermistors, an increase in electrical resistance translates to a decrease in skin temperature and vice versa [35].

Additionally, electrodermal activity (EDA) was recorded with skin resistance electrodes placed on the palmar surfaces of the distal phalanges of the third and fourth fingers of the contralateral hand only. Electrodermal activity was not recorded from the test hand for two reasons: (1) to avoid the interference of motion artifacts during the transitions between phases and (2) considering that a sympathetic influence on the VAR has been ruled out, our focus was to detect potential changes in sympathetic activity in the contralateral limb. Electrodermal activity signals are composed of two components: a slow-varying tonic component and a fast-changing phasic component [36]. Changes in the amplitude of the tonic component, as well as in the frequency and amplitude of the phasic component, reflect changes in cutaneous sympathetic activity, which are often evoked by cognitive or emotional stress. An increase in sympathetic-mediated sudomotor activity produces an increase in the transmission of electrical current through the skin. In addition, an increase in the EDA amplitude can also reflect an increase in muscle activity.

**Figure 2:**
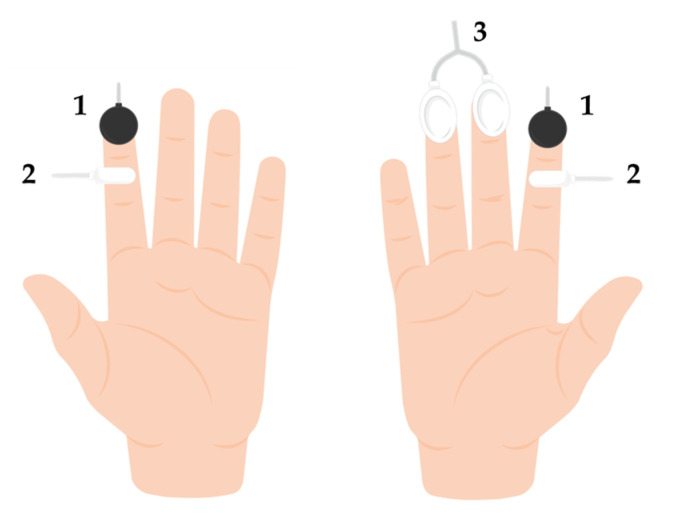
Schematic diagram of the experimental setup to illustrate the application of the PPG (1), temperature (2), and EDA (3) sensors to each hand (left—test; right—contralateral).

Figure 2 illustrates the application of the sensors to the test and contralateral hands. All sensors were connected to a BITalino Revolution^®^ microprocessor board (Biosignalsplux, Lisbon, Portugal). PPG and temperature sensors were connected to a 10-bit channel, whereas the EDA sensor was connected to a 6-bit channel. All signals were acquired at a 100 Hz sampling rate. In addition, blood pressure was measured with an automatic device (Omron X3 Comfort, Omron Healthcare, Kyoto, Japan) from the brachial artery at the heart level and 40 cm below the heart level.

### 2.4. Analytics

The following three periods were defined for statistical analysis: 4–6 min (baseline), 9–11 min (dependency), and 14–16 min (recovery). Skin blood flow was estimated as the amplitude of the PPG waveform (value at the systolic peak of a given pulse wave minus the value at the onset point of the following pulse wave) and was expressed in arbitrary units (AU). Pulse (min^−1^) was calculated as the number of PPG pulse waves per minute from the contralateral hand PPG signal. Skin temperature was expressed in degrees Celsius. Electrodermal activity was defined as the average value of the tonic component of the skin resistance signal and was expressed in microSiemens (µS). The temperature and EDA values were initially acquired by analog-to-digital converters and converted to their respective units according to the manufacturer’s specifications.

The median of the variation rate between the dependency and baseline (ΔII-I) was calculated for all variables and expressed as a percentage. Additionally, the minimum skin blood flow and the time to reach the minimum skin blood flow during dependency (t-min) were calculated for both hands. We observed a sharp increase in EDA during the transition between the baseline to dependency and between dependency to recovery. As such, the maximum EDA value and time-to-maximum EDA during the dependency (max-D; t_max-D_) and recovery (max-R; t_max-R_) phases were also calculated. The Shapiro-–Wilk test revealed that most variables were normally distributed. However, given the relatively small sample size, nonparametric tests were used to ensure analysis robustness. For each phase, all variables were defined as the medians and the corresponding limits of the 95% confidence intervals. Phase comparisons and limb comparisons were carried out using the Wilcoxon signed-rank test for related samples. For all statistical analyses, a 95% confidence level was used. Statistical analyses were performed with SPSS (version 21.0, IBM Corp, Chicago, IL, USA).

## 3. Results

**Figure 3:**
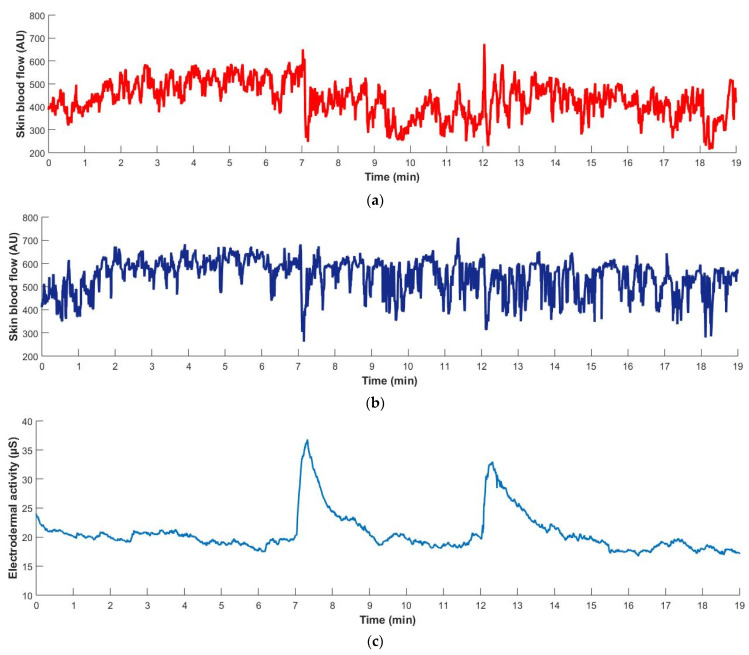
Median (*n* = 15) skin blood flow of the test hand (**a**, red) and contralateral hand (**b**, dark blue) and electrodermal activity (**c**, light blue) throughout the procedure. The baseline occurred from 0 to 7 min, dependency from 7 to 12 min, and recovery from 12 to 19 min.

The graphical representations of the evolution of median skin blood flow and median EDA are presented in Figure 3. The medians and 95% confidence intervals of the main variables in each phase of the procedure are presented in Table 2. During the baseline, no significant difference was detected in skin blood flow or skin temperature between limbs. When the test hand was placed below the heart level, skin blood flow decreased significantly in both hands, although more pronouncedly in the test hand (test: −19.6%, *p* = 0.003; contralateral: −6.7%, *p* = 0.005). During dependency, no significant differences were found in terms of median blood flow or minimum blood flow between limbs. Upon resuming the initial position, blood flow remained significantly lower than at the baseline in both limbs (test: *p* = 0.027; contralateral: *p* = 0.005), although the test limb showed a significantly lower blood flow (*p* = 0.012). Skin temperature did not change significantly either during dependency or during recovery. Similarly, the median electrodermal activity also did not change significantly throughout the protocol. Nevertheless, a peak in the EDA signal was noted during the transition between the baseline and dependency phases and between the dependency and recovery phases, after which the signal stabilized. The maximum EDA value of each peak was significantly higher than the baseline EDA value (max-D: *p* = 0.003; max-R: *p* = 0.004). However, no significant differences were detected between the maximum EDA value of each peak or between the time to reach each peak. Finally, the median pulse increased significantly during dependency, although the median relative change was null (*p* = 0.019).

## 4. Discussion

A major obstacle to the understanding of the precise mechanisms underlying the VAR is the considerable heterogeneity of experimental procedures that evoke this reflex. In fact, these studies differ in terms of the type (limb dependency vs. venous occlusion) and duration (1–10 min) of the applied maneuver, the subjects’ anatomical posture (sitting vs. supine), the anatomical location (upper vs. lower limb), the side of the body (right, left, or randomly chosen) and the tissue (skin, subcutaneous fat, or skeletal muscle) being assessed, the room temperature, the recording techniques employed (laser Doppler flowmetry or LDF, PPG, Doppler ultrasound, or ^133^Xenon washout), skin temperature (heated vs. not-heated), as well as the quantitative parameters assessed (blood flow, vascular conductance, vascular resistance, or vascular caliber). All these factors seem to influence the specific mechanisms that underlie the vasoconstrictive response to venous congestion, as well the magnitude of the perfusion reduction. In this section, we provide a critical comparison between the present results and those of previous publications and highlight the major sources of variability that hinder our knowledge of the VAR. Additionally, we provide novel insights into the underlying mechanisms of both the VAR and the contralateral microvascular response, with the aim of improving the conception of future studies.

In the present study, we report that unilateral upper limb dependency evokes a significant decrease in perfusion in both hands, test and contralateral, thus corroborating previous findings for the lower limbs [19,24]. During baseline, no significant differences were detected in the baseline skin perfusion and temperature between hands, meaning that we can assume equivalent hemodynamic conditions in both limbs. In our subjects, a 40 cm arm dependency caused a 19.6% decrease in perfusion in the test hand. Dependency increases the hydrostatic pressure in both venous and arterial territories [37], the latter being supported by a modest increase in blood pressure (Table 1). Therefore, the observed reduction in perfusion is likely explained by the simultaneous occurrence of VAR- and myogenic-mediated vasoconstriction [2,3].

Despite the high sensitivity of the NTC thermistors, no significant changes in skin temperature were detected in each limb during dependency. However, skin perfusion decreased gradually throughout the procedure and, during recovery, it failed to return to baseline values. This observation has been reported in previous studies [19,38]. However, since skin temperature was not controlled in those studies, a potential thermoregulatory-mediated vasoconstriction cannot be fully excluded. In the present study, however, no significant differences in skin temperature were detected between limbs in any phase of the procedure. Therefore, it is safe to assume that no thermoregulatory phenomena were responsible for the observed perfusion changes. As such, it stands to reason that the observed gradual decrease in perfusion is likely related to the immobilization of the upper limb. Limb immobilization may cause a decrease in tissue perfusion due to the decreased release of vasodilator tissue metabolites.

### 4.1. Influence of the Anatomical Location on the Venoarteriolar Reflex

Previous studies have shown a higher VAR magnitude in the arteries of the lower limbs than of the upper limbs using Doppler ultrasound [14,39]. This observation is consistent with the purpose of the VAR itself, which is to prevent the formation of edema due to increased venous pressure [40]. Below the heart level, vascular territories are subjected to increasing hydrostatic pressures the further they are from the heart [25]. Conversely, the further a vascular territory lies above heart level, the less it is affected by the hydrostatic pressure. Because the venous pressure is considerably larger in the lower limbs, the VAR displays a higher magnitude than in the upper limbs [1,39] or in the head and neck [1,41,42]. Another important determinant of the VAR magnitude is the density of perivascular innervation [43], which is presumably different between upper and lower limbs since it seems to depend on the venous pressure itself. Our results, together with those reported by Estañol-Vidal and colleagues (2018), are aligned with this physiological determinant. Both our studies assessed perfusion with PPG in the second finger of healthy subjects with a 40 cm hand dependency [22]. The more pronounced decrease in perfusion reported by those authors (35%) is explained by the use of a red light PPG sensor, which penetrates deeper into the skin and reaches vessels of a larger caliber when compared with our green light sensor [44]. Nevertheless, in both these studies, the VAR was considerably less intense than that reported by Silva and colleagues (2018) [19] and by Rodrigues and colleagues (2023) [24] during a 50 cm foot dependency (~90%), where perfusion was also recorded with a green light PPG sensor. Still, the considerable methodological differences between these studies (subjects’ age, female subjects’ menstrual cycle day, anatomical posture, and room temperature) may also influence the VAR magnitude.

In contrast, there are several studies showing no appreciable differences between upper and lower limbs or even showing the opposite trend. For example, in the studies by Okazaki and colleagues and Snyder and colleagues, the magnitude of the VAR was similar in the skin of the volar forearm and of the calf [2,40]. Also, Vissing and colleagues showed that leg/arm dependency from a supine position resulted in a more pronounced VAR in the forearm than in the calf [45]. There are three common denominators to these studies that may assist with the interpretation of this atypical finding:

- Skin microcirculation was assessed using LDF. It is known that optical technologies with different wavelengths reach skin at different depths and therefore assess different microvascular networks [44]. This has been consistently observed when comparing LDF with PPG [46,47], PPG of different wavelengths (green vs. red) [44], as well as LDF of different wavelengths [17];
- The assessed skin was heated to 34 °C. Analyzing heated skin may lead to a misrepresentation of skin blood flow during limb dependency. Heating such small skin areas does not change the blood distribution in the entire limb/region. Therefore, it is logical that the VAR-mediated vasoconstriction in heated skin may be less intense compared to the vasoconstriction in non-heated skin;
- Non-glabrous skin regions were assessed, which are known to have considerably less arteriovenous anastomoses (AAVs) and less post-ganglionic sympathetic fibers than the glabrous skin of the fingers and toes [48]. Therefore, it is possible that the magnitude and physiological mechanisms underlying the VAR differ between glabrous and non-glabrous skin.

### 4.2. Potential Involvement of the Sympathetic Nervous System in Upper Limb Dependency

Many studies have attempted to shed light on the involvement of the sympathetic nervous system in the VAR. Some studies reported that the VAR was abolished by sympathectomy and by the adrenergic blocker phentolamine [5,11,49]. In contrast, several other studies have reported that the VAR persisted following acute spinal and sympathetic neural blockade proximal to the measurement sites [4,10,12,45,50,51,52] following denervation in skin flaps [53] and in areas distal to nerve lesions in paraplegic [8,9] and tetraplegic patients [10]. While the explanation for the discrepancy in the effects of sympathectomy is still currently unclear, the VAR-blocking effect of phentolamine proposed earlier [5] was recently clarified to occur due to the vasodilatory effect caused by the high dose of that drug. In fact, more recent studies have demonstrated that the VAR was not affected by the application of adrenergic blockers. Crandall and colleagues demonstrated that the application of terazosin, yohimbine, or phentolamine did not affect the magnitude of the VAR of the upper limbs to dependency [3]. Even though post-ganglionic sympathetic fibers also co-release neuropeptide Y and ATP, both acting as cutaneous vasoconstrictors [54,55,56], that study also demonstrated that the VAR was unaffected by bretylium tosylate (an inhibitor of the release of post-ganglionic transmitters), thereby ruling out a predominant role for these transmitters. Snyder and colleagues reported that the intravenous administration of propranolol (i.e., a non-selective beta blocker) and phentolamine did not affect the magnitude of the VAR to either upper or lower limb dependency [14]. Taken together, these results demonstrate that the VAR is not mediated by sympathetic adrenergic nerve fibers.

In the present study, the subjects were naïve to the experimental procedure. As such, we assumed that sympathetic activation could occur due to a potential mental stress response, which would act as a confounding factor. For these reasons, EDA was quantified to discriminate the origin of any potential changes in sympathetic activity, whether directly related to the VAR or not. Median EDA values did not change significantly between the different periods of the signal analysis (Table 2). However, we noted the occurrence of transient but pronounced peaks in the EDA signal during the first minute of the dependency and recovery phases, as observed in Figure 3. These peaks correspond to phasic EDA, which translates to an increase in the cutaneous sympathetic activity. Once the hand was stabilized in each position, the EDA signal reverted to tonic activity. These peaks in sympathetic activity were not considered motion artifacts since the EDA electrodes were fixed to the contralateral limb, which was kept immobile throughout the procedure. Also, these peaks could not be attributed to a thermoregulatory response because skin temperature did not change significantly throughout the procedure. We also exclude the possibility of a baroreflex-mediated increase in sympathetic activity because there was no change in the anatomical position of the arteries of the trunk and upper extremities that could evoke this reflex [57]. The very discrete (2 bpm) increase in pulse during arm dependency confirms this explanation. The possibility of a mental stress response to the procedure itself was also excluded. Although our subjects were naïve to the procedure, none reported having perceived stress, including during changes of limb position. Furthermore, the very discrete increase in pulse also does not corroborate this hypothesis. As such, we conclude that both increases in sympathetic activity are a compensatory autonomic response to the postural changes themselves. In the present experimental conditions, however, the precise origin of this autonomic response cannot be identified.

### 4.3. Potential Involvement of Non-Sympathetic Mechanisms

It is possible that the dependency-induced VAR and the contralateral response may arise from the activation of stretch receptors in the walls of veins or arteries during congestion [58] or even from skeletal muscles or tendons during limb mobilization [59]. Interestingly, a non-vasomotion mechanism linking arterial distension to perivascular sensory fibers has been proposed [60]. According to this study, arterial distension causes the release of 20-hydroxyeicosatetraenoic acid, which acts on the transient receptor potential vanilloid 1 (TRPV1) channels present in perivascular sensory C fibers. In turn, these fibers release substance P, which acts on the vascular smooth muscle of the arterioles to induce vasoconstriction and enhance vasomotion. It is possible that the neural component of the VAR may use such mediators. Studies aiming at exploring the potential role of TRPV1 channels should also be conducted.

A recent study by Dayan and colleagues reported that an intravenous infusion of aminophylline attenuated the VAR in the forearm skin of healthy subjects when it was induced by venous occlusion but not when it was induced by limb dependency [61]. The authors assumed a partial involvement of adenosine receptors in the VAR. However, several mechanisms of action of aminophylline (i.e., adenosine receptor blocker and cAMP phosphodiesterase inhibitor) may be acting simultaneously and perhaps with opposite effects on the vasculature, especially since it was administered systemically. Therefore, studies with more controlled conditions are needed to confirm the involvement of adenosine receptors, their sites, and mechanisms of action.

Recently, Fujii and colleagues (2020) demonstrated that the magnitude of the VAR was attenuated by a calcium-mediated potassium channel blocker (tetraethylammonium) and potentiated by a voltage-gated potassium channel blocker (aminopyridine) in healthy male subjects [16]. The involvement of potassium channels had been previously theorized [62] to explain the frequent reports by Henriksen and colleagues that the VAR was abolished by high doses of phentolamine [50,51,52]. Indeed, phentolamine also antagonizes imidazoline receptors, including ATP-sensitive potassium channels [63], thus supporting previous findings. These results demonstrate that potassium channels in the vascular smooth muscle are involved in the VAR. Future studies should aim at further clarifying the cellular pathway in which these channels participate. Also, it would be interesting to clarify whether endothelial potassium channels (e.g., intermediate- and/or small-conductance calcium-activated potassium channels) are also involved in the VAR.

### 4.4. Potential Influence of the Endothelium

As a consequence of VAR-induced vasoconstriction, the endothelial shear stress is likely to decrease in the dermal capillary plexuses, which, hypothetically, could decrease the secretion of nitric oxide and, consequently, contribute to decrease perfusion. This potential attenuation of endothelial activity was also recently proposed by Silva and colleagues (2018) to partially justify the decrease in perfusion during lower limb dependency [19]. Davidson and colleagues (2004) also proposed the involvement of the endothelium in the VAR. These authors reported that the magnitude of the VAR was attenuated by the application of an inhibitor of nitric oxide synthase (i.e., N^G^-nitro-L-arginine methyl ester) but increased by an NO donor (i.e., sodium nitroprusside) in the heated skin of healthy subjects [34]. In contrast, Fujii and colleagues (2020) demonstrated that the endothelium plays no role in vasoconstriction during limb dependency in the non-heated skin of healthy male subjects [16]. In this study, the magnitude of the VAR was not affected by the application of inhibitors of the nitric oxide synthase (N^G^-nitro-L-arginine) or cyclooxygenase (ketorolac). However, because no female subjects were included in that study it is possible that an estrogen-mediated modulation of endothelial activity may have been overlooked [64,65].

### 4.5. Regarding the Decrease in Contralateral Perfusion 

In the present study, we report a significant decrease in contralateral perfusion (−6.7%) in response to unilateral upper limb dependency. This response is of considerably lower magnitude than the ones reported for the lower limbs by Silva and colleagues (2018) for young adults (−31%) [19] and by Rodrigues and colleagues (2023) for older adults (−23%) [24]. These differences suggest that the magnitude of the contralateral response is positively related to the magnitude of the hydrostatic pressure effect. The increase in sympathetic activity is the most likely explanation for the significant decrease in contralateral perfusion. As observed in Table 2, the median time to reach the first EDA peak and minimum contralateral perfusion after inducing dependency were 10 s and 13 s, respectively. This temporal overlap suggests that the increase in sympathetic activity is related to the reduced contralateral perfusion. Upon returning the test limb to the initial position, the second EDA peak was observed, together with the continuing decrease in contralateral perfusion. These observations, together with the fact that both EDA peaks occurred at similar times after each change in limb position, strongly suggest that the increase in sympathetic activity was evoked by the postural changes themselves, rather than the VAR, and induced contralateral vasoconstriction. Our results are in direct opposition to previous studies reporting that limb dependency does not significantly affect the perfusion of the contralateral limb. A study by Henriksen reported that leg dependency did not significantly affect the perfusion of the contralateral limb [11]. However, the small sample limits the interpretation of those results.

The neural pathways that control the peripheral vasculature are not well described, including the descending spinal pathways and their coordination with higher hierarchical structures [66,67]. Even though further studies must be conducted to clarify the origin of this contralateral response, some speculations are admissible. Spinal/supraspinal reflexes and/or local nonadrenergic responses have been proposed to occur simultaneously with the VAR [62]. Indeed, the consistent observations of a decrease in contralateral perfusion in the upper and lower limbs are apparently in favor of a “consensual” spinal reflex initiated by unilateral limb dependency that increases sympathetic activity toward the vasculature. In contrast to the VAR, where it was demonstrated that no brain integration occurs, the same does not necessarily apply to this contralateral response. Therefore, in order to assess whether the brain integration of venous afferent inputs is relevant, studies would need to be carried out in patients with spinal transection or equivalent animal models. Alternatively, inducing unilateral limb dependency with local drug application to the contralateral limb could also provide important results. For example, the blunting of the decrease in the contralateral perfusion of skin regions under the effect of a local anesthetic and of an adrenergic blocker would confirm that this response is indeed mediated by sympathetic adrenergic fibers.

### 4.6. Practical Implications of the VAR

The venoarteriolar reflex is an important physiological response that maintains vascular homeostasis and is known to become dysfunctional (either blunted or potentiated) in states of challenged homeostasis, as well as in diseased states and as a result of drug administration. The VAR was demonstrated to be potentiated in aged normotensive subjects and to be positively correlated with the morning blood pressure surge [39]. These results suggest that the VAR could advance our current knowledge of the pathophysiology of hypertension. In contrast, another study reported an attenuation of the VAR in aged subjects [68], although several important data were missing from this study (e.g., medical history and medications), which preclude a direct comparison with the former. The VAR is also reported to become dysfunctional in several diseases, including diabetes mellitus, peripheral artery disease (PAD), peripheral venous disease (PVD), lipedema, secondary Raynaud’s phenomenon, and dysautonomia. In patients with these conditions, the VAR can either be a cause of clinical manifestations or a consequence of the pathophysiological mechanisms that underlying them. The VAR is blunted in patients with diabetes (with or without peripheral neuropathy) [69,70,71], although a recent study showed no differences compared with healthy controls [72]. Once again, methodological differences between studies limit a more straightforward comparison. In patients with peripheral artery disease (PAD), peripheral venous disease (PVD) [73], lipedema [74], Raynaud’s phenomenon [75], and dysautonomia [76], VAR dysfunction can negatively affect the course of the disease. Blunting of the VAR leads to limb edema, which in turn is associated with several health problems, from limited mobility and flexibility, heaviness, discomfort or pain perception, and negative body image [77] to an increased risk of skin infections [78]. Conversely, VAR potentiation could play a part in the deterioration of cardiovascular function and create hemodynamic instability. Finally, the VAR may also be attenuated as an iatrogenic effect of different types of drugs (e.g., anti-hypertensive, hormones, gabapentinoids, and chemotherapeutic agents, among others) that are known to decrease arteriolar vasomotor tone [79].

In conclusion, assessing the integrity of the VAR could be a simple and practical approach to assess overall vascular function in different groups of patients to either monitor disease progression and/or the response to therapeutic strategies. Early detection of VAR dysfunction could contribute to optimizing therapeutic strategies, improving patient outcomes, and decreasing healthcare costs.

## 5. Limitations

The authors recognize three limitations of this study. Firstly, the relatively small sample and slightly asymmetric distribution preclude the discussion of sex differences in the magnitude of the VAR. Although such a discussion was not the aim of this study, our results revealed no significant differences in the measured variables between the sexes (not published). Nonetheless, it is possible that the magnitudes of the VAR and of the contralateral response might be influenced by sex. In fact, estrogen is known to potentiate the endothelial release of nitric oxide [80], which in turn could decrease the myogenic tone. However, in most of the previously published papers on the VAR, samples of similar size were used and no thorough discussion on the effect of sex was provided. Secondly, blood pressure was not measured continuously throughout the experimental procedure, and therefore a robust hemodynamic control could not be achieved, including the quantification of vascular conductance. Specifically, the lack of a continuous blood pressure measurement prevents the knowledge of whether the reduction in contralateral perfusion was attributed to local vasoconstriction or whether a decrease in blood pressure during limb dependency also occurred. As such, future studies should assess continuous blood pressure to gain insights into the mechanisms of the contralateral response. Thirdly, the fact that our subjects were naïve to the experimental procedure, having never performed it in the past, is likely to have acted as a confounding variable. Nevertheless, we provide evidence to support that the observed increases in sympathetic activity were not attributable to mental stress but rather to the postural changes. A future direction of research could be to explore the impact of mental stress on the magnitudes of the VAR and contralateral response.

## 6. Conclusions

This study has demonstrated that unilateral limb dependency evokes a bilateral decrease in perfusion in the hands of healthy subjects. The increase in hydrostatic pressure evokes the VAR and/or myogenic vasoconstriction, which decreases perfusion in the dependent limb. The magnitude of the perfusion decrease is considerably lower than that previously reported for the lower limbs, which are subjected to a larger hydrostatic effect. The observed contralateral response also supports previous findings, further suggesting the existence of a spinal/supraspinal reflex evoked by limb dependency that impacts contralateral limb hemodynamics. We propose that the decrease in contralateral perfusion evoked by limb dependency is attributed to an increase in sympathetic activity due to a postural change and not necessarily to venous/arterial congestion.

## Figures and Tables

**Table 1:** Characteristics of subjects (means ± standard deviations).

	Total	Females	Males
*n*	15	9	6
Age (years)	24.1 ± 5.8	23.0 ± 4.5	25.8 ± 7.5
Height (m)	1.71 ± 0.1	1.67 ± 0.1	1.77 ± 0.1
Body mass (kg)	63.8 ± 16.1	55.3 ± 8.1	73.8 ± 18.0
Body mass index (kg·m^−2^)	21.4 ± 2.9	19.8 ± 1.5	23.3 ± 3.2
Fasting duration (h)	2.1 ± 1.2	2.2 ± 1.1	2.1 ± 1.5
Mean menstrual cycle duration (days)		27 ± 1	
Menstrual cycle day		10.7 ± 7.8	
Systolic blood pressure (mmHg)			
At the heart level	109.6 ± 10.9	111.1 ± 11.4	107.4 ± 11.2
40 cm below the heart level	112.7 ± 13.8	113.4 ± 10.4	111.6 ± 18.9
Diastolic blood pressure (mmHg)			
At the heart level	66.8 ± 11.7	70.6 ± 10.3	61.6 ± 12.6
40 cm below the heart level	70.0 ± 13.2	72.9 ± 12.4	66.0 ± 14.8

**Table 2:** Medians and the limits of the 95% confidence intervals of the variables of the procedure (*n* = 15). Statistical comparisons between phases and between limbs were performed with the Wilcoxon signed-rank test for related samples (* *p* < 0.05).

	Test Hand	Contralateral Hand	*p* Value (Test vs. Contralateral)
**Skin blood flow (AU)**			
Baseline	547.0 (446.0; 663.7)	595.0 (433.0; 704.6)	0.436
Dependency	417.0 (177.7; 741.1)	570.0 (449.5; 701.8)	0.006 *
Recovery	410.0 (281.4; 684.2)	560.0 (464.7; 712.9)	0.012 *
Δ II-I (%)	−19.6 (−60.3; −1.5)	−6.7 (−18.1; 0.52)	0.008 *
Minimum flow	262.0 (92.9; 325.5)	356.0 (161.9; 552,5)	0.424
t-min (s)	15.0 (9.3; 39.8)	9.0 (7.0; 39.3)	0.263
*p* value (Dependency vs. Baseline)	0.003 *	0.005 *	-
*p* value (Recovery vs. Baseline)	0.027 *	0.005 *	-
**Skin temperature (°C)**			
Baseline	31.4 (29.9; 34.4)	31.8 (31.5; 34.9)	0.636
Dependency	31.7 (30.1; 33.9)	32.0 (31.6; 35.0)	0.526
Recovery	32.0 (30.3; 33.9)	32.3 (31.3; 34.7)	0.626
Δ II-I (%)	−1.5 (−8.9; 2.4)	0.9 (−0.6; 1.5)	0.423
*p* value (Dependency vs. Baseline)	0.593	0.476	-
*p* value (Recovery vs. Baseline)	0.221	0.322	-
**Pulse (min^−1^)**			
Baseline	70.0 (61.7; 95,3)		-
Dependency	72.0 (56.6; 90.2)		-
Recovery	70.0 (57.5; 87.7)		-
Δ II-I (%)	0.0 (−1.3; 2.7)		-
*p* value (Dependency vs. Baseline)	0.019 *		-
*p* value (Recovery vs. Baseline)	0.301		-
**Electrodermal activity (µS)**			
Baseline	17.0 (7.2; 29.5)		-
Dependency	15.0 (13.2; 26.5)		-
Recovery	19.2 (12.0; 27.8)		-
Δ II-I (%)	−3.4 (−21.2; 72.4)		-
max-D	46.0 (30.3; 66.1)		-
t_max-D_ (s)	10.0 (5.9; 18.1)		-
max-R	47.0 (28.0; 62.8)		-
t_max-R_ (s)	13.0 (5.9; 16.5)		-
*p* value (Dependency vs. Baseline)	0.722		-
*p* value (Recovery vs. Baseline)	0.638		-
*p* value (max-D vs. Baseline)	0.003 *		-
*p* value (max-R vs. Baseline)	0.004 *		-
*p* value (max-D vs. max-R)	0.066		-
*p* value (t_max-D_ vs. t_max-R_)	0.341		-

## Data Availability

The data presented in this study are available upon request from the corresponding author.
